# Proving a negative? Methodological, statistical, and psychometric flaws in Ullmann et al. (2017) PTSD study

**Published:** 2018-03-25

**Authors:** Gregory J. Boyle

**Affiliations:** University of Melbourne, Australia

**Keywords:** PTSD, circumcision, negative results, underpowered studies, null-hypothesis significance testing

## Abstract

Ullmann et al. recently published a pilot study in Translational Psychiatry in which they report failing to find a statistically significant reduction in either hair cortisol or hair cortisone levels in circumcised men as compared with genitally intact (noncircumcised) men. Based on such null findings, the authors purport to have "refuted the psycho-pathological long-term effects of circumcision" and that the lack of significant results, "add to the growing body of evidence in the literature that male circumcision is not likely psychologically traumatizing across the life-span." In addition, they claim that they have proven a "healthy functionality of the LHPA axis" in men subjected to circumcision during infancy or childhood. However, it is not possible to draw any such conclusions on the basis of a null finding, especially one derived from an underpowered study in which the trend in the data suggest, if anything, that an adequately powered study may have shown the opposite of what the authors claim.

**Relevance for patients:**

When combined with other weaknesses in study design, measurement, and interpretation, it becomes apparent that the authors' conclusions are not supported by their data.

## 1. Introduction

The issue of how to evaluate studies with negative findings has proven to be problematic and no satisfactory consensus has been reached to date. Nevertheless, there are many cases in which it is clearly inappropriate to draw substantive conclusions on the basis of a lack of statistically significant effects. Consider a recent pilot study by Ullmann et al. published in *Translational Psychiatry* in which the authors reported failing to find a statistically significant reduction in either hair cortisol or hair cortisone levels in circumcised men as compared with genitally intact men [1]. Based on such null findings, the authors claimed to have "refuted the psycho-pathological long-term effects of circumcision" and asserted that the lack of significant results "add to the growing body of evidence in the literature that male circumcision is not likely psychologically traumatizing across the life-span." In addition, they claimed to have proven a "healthy functionality of the LHPA axis" in men subjected to circumcision.

These are strong claims. Yet as will be demonstrated, it is neither logically nor statistically sound to draw any such conclusions on the basis of a null finding [2-4], especially when this finding is derived from an underpowered cross-sectional study in which the trend in the data suggest, if anything, that an adequately powered study may have shown exactly the opposite of what the authors claimed [5]. Indeed, as Ullmann et al. themselves reported [1].

*In uncircumcised subjects, concentration of Cortisol was 7.4 ± 1.4 s.e. pg mg-1 (N = 11) and cortisone 17.3 ±3.8 s.e. pg mg-1 (N = 10), whereas in circumcised subjects, concentration of cortisol was 5.7 ± 0.9 s.e. pg mg-1 (N = 9) and cortisone 14.2 ±1.2 s.e. pg mg-1 (N = 9).*

The aim of the present paper is to evaluate the study from Ullmann et al., treating it as a cautionary tale for researchers as to how *not* to interpret negative findings using the Null Hypothesis Significance Testing (NHST) approach [6].

**Study overview**

In order to assess long-lasting psychological trauma and ongoing stress (PTSD-like symptoms) among adult males subjected to genital cutting, Ullmann et al. measured objective hair cortisol and cortisone levels [7,8] and administered five introspective (subjective) self-report personality-stress questionnaires (see Box 1, "Lack of psychometric sophistication" for a description) to a small sample of 20 self-selected circumcised and genitally intact men who had immigrated to Germany from the former USSR. In order to avoid "post-war psychosocial transgenerational transmission influences as well as acculturation effects" related to stress, men whose parents had been born before the end of WWII were excluded from the study. Also excluded were men with a history of "Cushing's disease, Addison's disease, hypo-/hyperthyroidism or other endocrine disorders." Ullmann et al. reported that, in relation to circumcision status, they found "no differences in long-term limbic-hypothalamic-pituitary-adrenal axis activity, subjective stress perception, anxiety, depressiveness, physical complaints, sense of coherence and resilience" [1]. They concluded that their negative findings provided evidence that "male circumcision does not promote psychological trauma" [1].

## 2. Main weakness of study

### 2.1. Methodological shortcomings

In their study, Ullmann et al. relied on a ('single-shot') cross-sectional between-groups design based on a convenience sample without stratified random allocation of participants within each of the two respective groups [2]. Such a static-group comparison design has well-documented sources of internal invalidity (especially selection, mortality, and the selection x mortality interaction), as well as external invalidity (selection x treatment interaction), which must be taken into account [9]. Among other concerns, this pre-experimental design allows "no formal means of certifying that the groups [are] equivalent" [9]. And yet, aside from asking about the men's involvement in sporting activities and sociodemographic background information (age, education, income, hair washing frequency, cosmetic hair treatment, age at circumcision), there was little attempt to control for differences between the two comparison groups on multiple unmeasured background variables, any of which potentially could have operated as confounders to contaminate the findings[10-12]. The most serious error committed by Ullmann et al., however, concerns their unsupported inference from a lack of statistical significance to proof of the null hypothesis that there is no difference in PTSD-like symptoms in relation to men's circumcision status. This error alone is enough to invalidate the study by Ullmann et al., as described in the following sections.

### 2.2. Statistical shortcomings

While the authors conducted conservative, two-tailed t-tests (without Bonferroni correction) on each of the multiple dependent measures, there was justification for conducting more sensitive one-tailed t-tests to assess the hypothesis that circumcised men might exhibit significantly higher levels of PTSD-like symptoms than genitally intact men. Using a two-tailed t-test to ' refute' a directional hypothesis may result in failure to find a significant effect when there really is a difference in the population (Type II error). Also, the smaller the sample size, the greater is the likelihood of a Type II error. Relevant for the Ullmann et al. study, "failure to reject the null hypothesis does not imply that the null hypothesis is true [but] many investigators exhibit an inclination to conclude, even for quite small samples, that no difference, or a trivial difference, exists when a required level of significance is not achieved ... such conclusions are unwarranted"[13]. Regrettably, many researchers "are tempted to conclude [that] they have in effect 'proved' that the null hypothesis is true [even when] the experiment is not sufficiently sensitive to detect [actual differences]" [4].

**Box 1.** Lack of psychometric sophistication

The five scales used were the 30-item Perceived Stress Questionnaire (PSQ) [14,15], the 24-item short form of the Giessen Subjective Complaints List (GBB-24) [16], the 14-item Hospital Anxiety and Depression Scale (HADS) [17,18], the abbreviated 9-item Sense of Coherence (SOC-9L) Scale [19,20], and the 13-item German version of the Resilience Scale (RS-13) [21,22]. The authors' reliance on subjective self-report questionnaires was less than ideal given the possibility of socially desirable responding or other forms of motivational and response distortion [23-26]. The authors claimed that the subjective self-report scales met the "highest national and international quality standards" citing high Cronbach alpha coefficients (ranging from 0.80 to 0.93) as evidence. However, such high levels of intra-scale item homogeneity are potentially problematic and say nothing at all about the temporal consistency or psychometric validity of the scales used [27]. In contrast to the authors' sweeping assertions about the positive psychometric properties of the self-report scales, in fact, they failed to provide any evidence of test-retest reliability of the scales over time (neither dependability nor stability coefficients were reported) [28], nor was any evidence provided of factor analytic, construct, discriminant, concurrent, or predictive validity, as per standard psychometric reporting requirements [29-31]. Furthermore, Ullmann et al. provided no evidence of having counterbalanced the order of administration of the respective scales, thereby failing to control for possible position effects [9,32]. Given these multiple problematic issues, the reported failure to find a statistically significant difference between circumcised and genitally intact men on any of the five personality-stress measures remains inconclusive with respect to their study hyp othesis.

#### 2.2.1 Lack of power

The NHST procedure used by Ullmann et al., while commonly employed, has been strongly criticized by statistical experts, with some authors arguing it is invalid [3]. But even those who do support the use of NHST in limited circumstances contend that adequate sample sizes are necessary for drawing justified conclusions about the implications of the data [33]. In light of this, it is concerning that just 11 genitally intact men were compared with 3 men circumcised as minors without analgesia (plus 6 men circumcised with analgesia). That is, fully two-thirds of the men included in the circumcised group had received analgesia, plausibly reducing (1) the likelihood of subsequent PTSD-like symptoms in this sub-group and thereby, (2) the chance of finding any significant between-group differences. This is a serious confounder that unnecessarily increased the minimum sample size of the "mixed circumcision" group required to observe any between-group significant differences on the dependent questionnaire measures.

As advised by methodologists Szucs and Ioannidis, any researchers who do choose to use the NHST approach, despite its shortcomings, must "justify its use, and publish pre-study power calculations and effect sizes" wherever feasible [33]. Since Ullmann et al. used NHST to reach the negative conclusion that circumcision produced no long-lasting PTSD-like symptomatology, it was especially incumbent on them to have performed such calculations. If they had done so, they would have found that the very small sample size of each comparison group was insufficient to have demonstrated any significant between-group differences on any of the questionnaire measures, even if such differences existed within the population. Given much larger sample sizes and adequate power, clinically relevant differences may well have been observable on the multiple dependent self-report questionnaire measures.

In order to determine the minimum sample sizes needed in order to find any significant between-group differences, power analyses were conducted by the present author using two entirely different methods: the classical approach advocated by Cohen [34,35], and the more recent approach advocated by Trafimow that involves use of inferential statistics prior to data collection [36]. In the latter approach, the closeness of group means to their corresponding population means is specified, along with the level of confidence desired [37].

#### 2.2.2 Traditional Cohen method

A power calculation using Cohen's method (computed via G*Power 3.1) with moderate effect size = 0.50, power = 0.80, and a = 0.05, indicates that even without Bonferroni correction for the multiple dependent measures, at least 51 genitally intact men and 153 men in the 'mixed' circumcision group would be needed in order to have an 80% chance of observing any significant between-group differences on any of the dependent variables [38]. When a Bonferroni correction is applied, at least 82 genitally intact men and 246 men in the "mixed" circumcision group would be required to find any significant differences. If the power is increased to 0.95 (with moderate effect size), a minimum of 88 and 264 men would be required in the two groups (with Bonferroni correction, 128 and 384 men are needed, respectively). Assuming a small effect size = 0.20, power = 0.80, and a = 0.05, no fewer than 310 genitally intact men and 930 men in the "mixed circumcision" group would be required. With Bonferroni correction, 504 and 1512 men would be required in the two groups, respectively. If the power is increased to 0.95 (with small effect size), a minimum of 542 and 1626 men would be required in the two groups (with Bonferroni correction, 790 and 2370 men, would be needed, respectively). Thus, regardless of the presumed effect size, it is clear that the Ullmann et al. study was vastly underpowered.

#### 2.2.3 Trafimow's method

Trafimow has recently introduced a novel means of estimating the necessary minimum sample size required for a valid experiment [36]. This radically different estimation method provides the necessary per-group sample size (n and n2) by computing Equation 1 prior to data collection, whereby $ is the cumulative distribution function *(cdf)* of the standard normal distribution (for expediency, rather than write out the integral of the normal equation, the Greek letter $ is used to designate an area under the standard normal curve), / is the desired precision (i.e., the goal is to have the sample mean be within / standard deviations from the population mean - the standard deviation fraction that the researcher defines as "close"), *k* is the number of comparison groups, and *n* is the requisite sample size [37]. The variables $, /, k, and the probability that sample means are within the specified distance, *p(k* Means), all act conjointly to influence the minimum sample sizes needed. The goal of Equation 1 is to obtain a sample mean that is within a specified distance from the population mean. While in traditional power analysis, the effect size strongly influences estimation of the requisite sample size, it plays no role whatsoever in computation of the Trafimow procedure [38]. Equation 1 allows estimation of the minimum sample size needed to meet the specifications concerning closeness and confidence, irrespective of the number of comparison groups.

**Figure:**
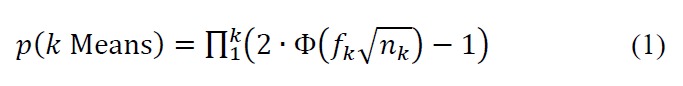


According to Trafimow, "With two groups, the total sample sizes needed are 48, 84, 186, and 742, when / = 0.4, 0.3, 0.2, or 0.1 [standard deviation units], respectively." Thus, "a desire for stringent precision . necessitates large samples [in order] to have a respectable probability" of obtaining replicable findings [39]. Even accepting the most favorable assumption (with / = 0.4 in the Ullmann et al. study), there would still need to have been at least 72 men in the mixed circumcision group and 24 men in the genitally-intact group. With / = 0.3, there would need to have been at least 126 men in the mixed circumcision group and 42 men in the genitally-intact group; with / = 0.2, there would need to have been at least 558 men in the mixed circumcision group and 93 men in the genitally-intact group; and with / = 0.1, there would need to have been at least 1113 men in the mixed circumcision group and 371 men in the genitally-intact group. This then provides still further evidence on statistical grounds alone that the sample sizes employed by Ullmann et al. were entirely inadequate to justify drawing any valid inferences whatsoever.

As Keppel stated, "it is not sufficient simply to fail to reject the null hypothesis to 'prove' it, but you must do so under conditions of high power . an experiment that is specifically designed to prove the null hypothesis usually requires a huge commitment of subjects" [4]. Since the sample sizes employed by Ullmann et al. were many times smaller than those required on a range of plausible effect size and power estimates, the observed null findings are uninterpretable.

#### 2.3.4. Confidence intervals

We have seen that Ullmann et al. attempted to draw inferences about PTSD-like symptoms in relation to circumcision status on the basis of inadequate sample sizes and negative findings. Without considering possible confounding of sampling precision, homogeneity precision, and measurement precision [40,41], Ullmann et al. provided (unspecified) graphically-presented confidence intervals for cortisol and cortisone levels (Ullmann et al. Fig. 1). To provide greater accuracy and interpretability, the present author computed both the 95% and 99% confidence intervals for the reported cortisol and cortisone data (in pg mg-1), all of which appear to have been measured reliably, as follows:

Cortisol (11 genitally intact men):

95% CI: 7.4 ±2.7 (4.70 to 10.10); and 99% CI: 7.4 ±3.6 (3.80 to 11.00). Cortisol (9 circumcised men):

95% CI: 5.7 ±1.8 (3.90 to 7.50); and 99% CI: 5.7 ± 2.3 (3.40 to 8.00). Cortisone (10 genitally intact men):

95% CI: 17.3 ±7.4 (9.9 to 24.70); and 99% CI: 17.3 ±9.8 (7.50 to 27.10). Cortisone (9 circumcised men):

95% CI: 14.2 ±2.4 (11.80 to 16.60); and 99% CI: 14.2 ±3.1 (11.10 to 17.30).

However, Ullmann et al. omitted to report confidence intervals for any of the five subjective self-report questionnaires. Examination by the present author of both the 95% and 99% CIs reveals that the confidence intervals for the 30-item *Perceived Stress Questionnaire* scores (with only 7 circumcised men included since two PSQ response forms were incomplete; see Ullmann et al., Table 2) were unacceptably wide, showing that the mean PSQ scores reported were unreliable. The respective confidence intervals are as follows:

PSQ (7 circumcised men):

95% CI: 0.02 ± 0.16 (-0.14 to 0.18); and 99% CI: 0.02 ± 0.20 (-0.18 to 0.22).

Since Ullmann et al. stated that, "The PSQ meets the highest national and international quality standards" [1], one can only conclude that the sample size of 7 circumcised men was completely inadequate to obtain reliable PSQ scores. How many of these men had undergone circumcision without analgesia and how many with it? If most of them had received analgesia, then that would only have served to suppress any observed differences in PSQ scores between the circumcised and genitally intact groups, unduly favoring the null hypothesis.

Contrary to the authors' assertions that "circumcision does not alter long-term glucocorticoids' accumulation" (and despite not being significant due to insufficient sample size), the reported data from the Ullmann et al. study actually did show substantial reductions in circumcised men of 23% and 18% in objectively measured hair cortisol and cortisone levels, respectively, thereby making it difficult to rule out dysfunction of the limbic-hypothalamic-pituitary-adrenal (LHPA) axis. But, only an adequately powered study could properly answer the research question that Ullmann et al. had put forward.

## 3. Conclusion

The Ullmann et al. paper exhibits weaknesses beyond those described in the present analysis, including an overt misrepresentation of policy statements bearing on the subject of their investigation (see Box 2). What is most fundamentally at issue, however, is the authors' unjustified assertion of having proven the null hypothesis with an obviously underpowered study. It is not possible to ' prove' the null hypothesis, but only to fail to reject it [2-4]. Such misuse of the word ' prove' undermines the legitimacy of that term in scientific discourse. Caution is therefore warranted in interpreting null results. Absence of evidence-especially when that absence comes from an underpowered, small-sample-size study using a subset of measurement scales with discernible psychometric weaknesses (Box 1)-is not evidence, much less conclusive evidence that, "male circumcision is not likely psychologically traumatizing across the life-span." The conclusions drawn by Ullmann et al. are unsupported by their data.

**Box 2.** Misrepresentation of policy statements

For unclear reasons, Ullmann et al. erroneously claimed that the American Academy of Pediatrics (AAP) and the Centers for Disease Control and Prevention (CDC) "strongly recommend circumcision to promote hygiene and prevent disease." In fact, the most recent AAP policy explicitly did not recommend circumcision, stating that: "[The] health benefits are not great enough to recommend routine circumcision for all male newborns" [42]. The CDC, which released a non-peer-reviewed draft policy that has never been finalized or formally published, only recommended counseling [43-44]. Finally, the authors failed to mention that all other (international) peer organizations to the AAP that have released policies on newborn circumcision similarly do not recommend the procedure and have in addition concluded that the benefits do *not* outweigh the risks [45].

Moreover, despite the negative findings of Ullmann et al., the documented empirical evidence demonstrates unequivocally that circumcision (especially without analgesia) is highly stressful for infants and children and can be traumatic [46-49]. Gunnar and colleagues found that circumcision elicited more behavioral distress and evoked a larger cortisol response than blood sampling, weighing, or physical examination [50]. When asked, nurses and physicians ranked circumcision (along with chest tube insertion) as the most painful of all procedures performed in the neonatal intensive care unit [51].

Although individual differences in personality trait characteristics (such as resilience) suggest that the same potentially traumatic incident may impact on individuals differently [52], available reports of PTSD-like symptoms from many circumcised men-some of whom directly associate long-lasting symptoms with their genital surgeries and/or resultant adverse psychosexual effects-suggest that further careful research into PTSD-like symptoms pursuant to circumcision is warranted [53-65].

In the context of this discussion, it is important to recognize that negative results may sometimes be meaningful, and scientific journals do need to be more open to publishing such results as argued recently in this journal [66]. But in order for null findings to tell us anything, they need to be derived from well-designed, well-controlled, well-conducted experiments with sufficient power and adequately large sample sizes to enable the detection of meaningful effects should they actually exist in the population, which are then replicated in subsequent cross-validation studies [67]. In light of such stringent requirements for interpreting null findings, it is evident that the study by Ullmann et al. was plagued by serious methodological, statistical, and psychometric flaws that the authors appear to have overlooked, suggestive of substantial experimenter bias[68-72].
